# Prevalence, clinical characteristics and echocardiographic parameters of arrhythmias among patients with rheumatic heart disease attending Jakaya Kikwete Cardiac Institute: a prospective cohort study

**DOI:** 10.1186/s12872-023-03427-0

**Published:** 2023-08-17

**Authors:** Clara Damascene Makatu, Reuben Kato Mutagaywa, Ponsian Peter, Aileen Barongo, Engerasiya Kifai

**Affiliations:** 1 School of Medicine, Department of Internal Medicine, Muhimbili University of Health and Allied Sciences, Dar Es Salaam, P.O.BOX 65001, Tanzania; 2 Department of Adult Cardiology, Jakaya Kikwete Cardiac Institute, Dar Es Salaam, Tanzania; 3 School of Medicine, Department of Pharmacology, Muhimbili University of Health and AlliedSciences, Dar es salaam, Tanzania; 4 Department of Pediatrics and Child Health, Mwananyamala Regional Referral Hospital, Dar es Salaam, Tanzania

**Keywords:** Rheumatic heart disease, Electrocardiography, Left atrium, Heart failure, Mitral stenosis, Arrhythmias

## Abstract

**Background:**

Arrhythmia is a known complication of rheumatic heart disease (RHD). It is critical to recognize arrhythmias early on so that prompt preventative actions and vigilant monitoring may be considered while treating these patients.

**Aim:**

This study aimed at determining the prevalence, clinical characteristics and echocardiographic parameters of arrhythmias among RHD patients attending Jakaya Kikwete Cardiac Institute (JKCI).

**Methodology:**

Hospital-based cross-sectional study was conducted among 390 patients with an echocardiographic diagnosis of RHD aged 18years and above attending JKCI. Demographic and clinical information was documented. Echocardiography, a resting electrocardiography and 24 h ambulatory Holter monitoring electrocardiography were done. Chi square test was used to determine association between variables and those with a p value ≤ 0.2 were entered in a multivariate logistic regression analysis to determine the independent factors associated with arrhythmias. P value of < 0.05 was considered statistically significant. The receiver operating curve was used to determine the critical point for left atrial size beyond which arrhythmias develop.

**Results:**

A total of 390 patients were included in the analysis. The median age was 39 years interquartile range (IQR 30–52). Females were 257/390 (65.9%). Asymptomatic patients were 208/390 (53.3%). Most patients belonged to New York heart Association (NYHA) functional class I&II 247/390 (62.1%). The most common valve lesion was mitral stenosis 228/390 (58.5%). Arrhythmias were found in 276/390 (70.77%) patients, of which 193/390 (49.5%) patients were from resting electrocardiography (ECG) and 88/197 (44.7%) patients from holter ECG. Independent factors for arrhythmias were, NYHA functional class III&IV (a0R 4.67, 95% CI 1.82-12.00 p = < 0.01) and severe left atrial diameter enlargement (aOR 7.28, 95% CI 3.17–16.70 p = < 0.01). The critical point beyond which arrhythmias develop was found to be left atrium diameter > 48 mm.

**Conclusion:**

We found a high prevalence of arrhythmias among patients with RHD. The independent predictors of arrhythmias were left atrium dilatation and NYHA functional class III-IV. We recommend close monitoring for arrhythmias among RHD patients in sinus rhythm with higher NYHA functional class and dilated left atrium.

## Background

Rheumatic heart disease results from valvular damage secondary to an abnormal autoimmune reaction after infection with group A streptococcal (GAS) bacteria. This disease has disappeared in developed countries but is still a major problem in the third world countries particularly with low social economic status [1, 2]. Socioeconomic conditions leading to increased GAS exposure include household crowding, poor hygiene, and low access to medical services [3]. RHD is a prevalent trigger of arrhythmia, but its frequency and concomitant arrhythmias have been steadily declining in Western countries in recent years. However, in both emerging and third-world nations, the frequency remains high [4].

A study done in India found that out of 84 patients with RHD, atrial fibrillation (AF) was present in 31(37%) on baseline ECG. Thirteen out of 53 patients who had sinus rhythm on baseline ECG were found to have Paroxysmal AF on 24-hours Holter ECG monitoring. Premature ventricular contractions (PVCs) were present in 72(85.7%), those with couplets where 52(62%), bigeminy was 25(29.7%) and trigeminy 17(20.2), pauses were 39(46.7%) on holter[5]. This indicates the importance of close monitoring of these patients and further investigating for arrhythmias by 24-hour holter monitoring even when resting ECG is not suggestive of arrhythmias.

Echocardiography plays a significant role in the evaluation of patients with arrhythmias because it offers information on structural alterations favoring arrhythmogenesis, risk stratification, and therapeutic decisions. Arrhythmia pathophysiology in RHD has been linked to structural alterations involving the heart [6].

In a study done in India on arrhythmias in valvular heart disease which predominantly comprised of rheumatic heart disease patients, they established a relationship between echocardiographic parameters with arrhythmia [7]. It was found that the critical point beyond which the patient is more prone to get an arrhythmia was when Left atrium (LA) diameter > 43 mm, mitral valve area (MVA) < 1.7cm^2^, mitral MG (mean gradient) > 9 mm Hg, Left ventricular internal diameter in diastole (LVID D) > 50 mm and Left ventricular internal diameter in systole (LVID S) > 39 mm. Patients with multivalvular involvement were more prone to arrhythmia [8]. In another study AF was associated with LA diameter of greater than 40 mm (OR = 7.5, CI 2.4–9.8, p = 0.001) [9]. Also, in a cohort study done in Ethiopia AF among RHD patients was associated with LA size of greater than 45 mm [7].

Although RHD is still a major problem in developing countries it seems to be a matter of indifference, and little is known about RHD and most of what is known has been derived from studies done thirty years ago. Understanding the pathophysiology of the disease and its sequalae is an important aspect towards management.This studyaimed at determining the prevalence, clinical characteristics, and echocardiographic findings of arrhythmias among patients with RHD.

## Methods

This was hospital-based cross-sectional cohort study done at Jakaya Kikwete Cardiac Institution (JKCI), Dar-es-salaam, Tanzania. All patients with Echocardiographic diagnosis of RHD aged 18 years and above were consecutively recruited until a sample size of 390 was reached. The study was conducted from June to December 2021.Cases eligible to participate in the study were included only after being provided with informed consent.

### Study procedures

Clinical forms and structured questionnaire were used to obtain the patients demographic and clinical characteristics. Physical examination was done to all patients and all findings were recorded in the clinical forms; patients were classified according to the NYHA based on their symptoms. Echocardiography was done to all patients using the SC 2000 S ECHO machine. Electrocardiography was also perfomed to all patients using the General electronic Mac400machine. Patients who had a normal sinus rhythm on resting ECG also performed a 24-hour Holter ECG monitoring in a range of 0 to 4 days after the echocardiographic examination. Results obtained were recorded in a clinical form. The echocardiogram examination, chamber dimensions and quantification followed the American Society of echocardiography guidelines [10, 11].

#### Operational definitions

Arrhythmias on 24 h HolterECG were defined as follows [12]: Ventricular ectopic; >15ectopic beats per 1,000 ventricular beats; Supraventricular ectopic: >15 ectopic beats per 1,000 atrial beats; Paroxysmal AF: an irregular rhythm without P-wave activity sustained for ≥ 10 beats; Paroxysmal atrial flutter: presence of flutter p wave activity sustained for > 10 beats, and Non-sustained ventricular tachycardia was defined as a broad complex tachycardia sustained for > 5 beats. If sustained for ≥ 30 s, the arrhythmia was termed as sustained ventricular tachycardia (VT).

### Data analysis

Data was entered on SPSS version 23 for statistical analysis. Categorical variables were presented as frequencies and percentages, continuous variables were presented as median and IQR. Chi square test was used to establish the association between social-demographic, clinical characteristics and echo parameters with arrhythmias. All factors with p value < 0.2 were entered into a multivariate logistic regression analysis to determine the independent echocardiographic factors associated with arrhythmias, their adjusted odds ratio (aOR), 95% confidence interval and p-value were determined. P value < 0.05 was considered statistically significant. Receiver operating Curve was used to determine the cutoff point beyond which arrhythmias developed.

### Ethical consideration

Ethical clearance to conduct the study was obtained from Muhimbili University of Health and Allied Sciences’ Ethical Review Board. Permission to do the study was obtained from JKCI management. Informed consent was obtained from all study participants before they were enrolled in the study.

## Results

### Socio-demographic and clinical characteristics of the study population

A total of 400 patients with echocardiographic diagnosis of RHD were recruited during June – December 2021, 7 did not report on their scheduled day for 24-Holter monitoring, 3 denied consent. The final population consisted of 390 patients. Median age was 39 years IQR (30,52), majority were female 257/390 (65.9%), while 202/390 (51.8%) attained primary education. The median BMI was 23 kg/m^2^ (IQR 14.3–47.6), 373/390 (95.6%) had no co-morbidities, 275/390 (70.5) had history of cardiac surgery. We found that 208/390 (53.3%) were asymptomatic for arrhythmias, most patients were in NYHA class I and II by 242/390 (62.1%)and 119/390 (30.5%) respectively. The commonest valve pathology was mitral stenosis by 228/390 (58.5%) as shown in Table 1.

**Table 1:** Socio-demographic characteristics of the study participants, n = 390

Variables		n = 390	
Age group (years)			
	18–30	101 (25.9)	
	31–50	181 (46.4)	
	51–60	57 (14.6)	
	> 60	51 (13.1)	
Median age in years (IQR)		39 (30,52)	
Sex			
	Male	133 (34.1)	
	Female	257 (65.9)	
Level of education			
	Primary	202 (51.8)	
	Secondary school	137 (35.1)	
	College / University	51 (13.1)	
BMI (kg/m^2^)			
Underweight		28 (7.2)	
Normal		225 (57.7)	
Overweight		69 (17.7)	
Obese		68 (17.4)	
Median BMI (in kg/m^2^)		23 (14.3,47.6)	
Comorbidities			
Yes		17 (4.4)	
No		373 (95.6)	
History of cardiac surgery			
Yes		98 (25.1)	
No		275 (70.5)	
Symptoms of arrhythmias			
Symptomatic		182 (46.7)	
Asymptomatic		208 (53.3)	
NYHA functional class			
I-II		242 (62.1)	
III-IV		148 (37.9)	
Valve lesion			
Mitral stenosis		228 (58.5)	
Mitral regurgitation		184 (47.2)	
Aortic stenosis		27 (6.9)	
Aortic regurgitation		126 (32.3)	
Tricuspid regurgitation		187 (47.9)	

Data are expressed as n (%) or median (IQR) where appropriate. n: Number of patients, IQR: Interquartile range, BMI: body mass index was calculated using height and weight (BMI = weight in kg /height in meters2), NYHA: New York Heart Association.

**Fig. 1:**
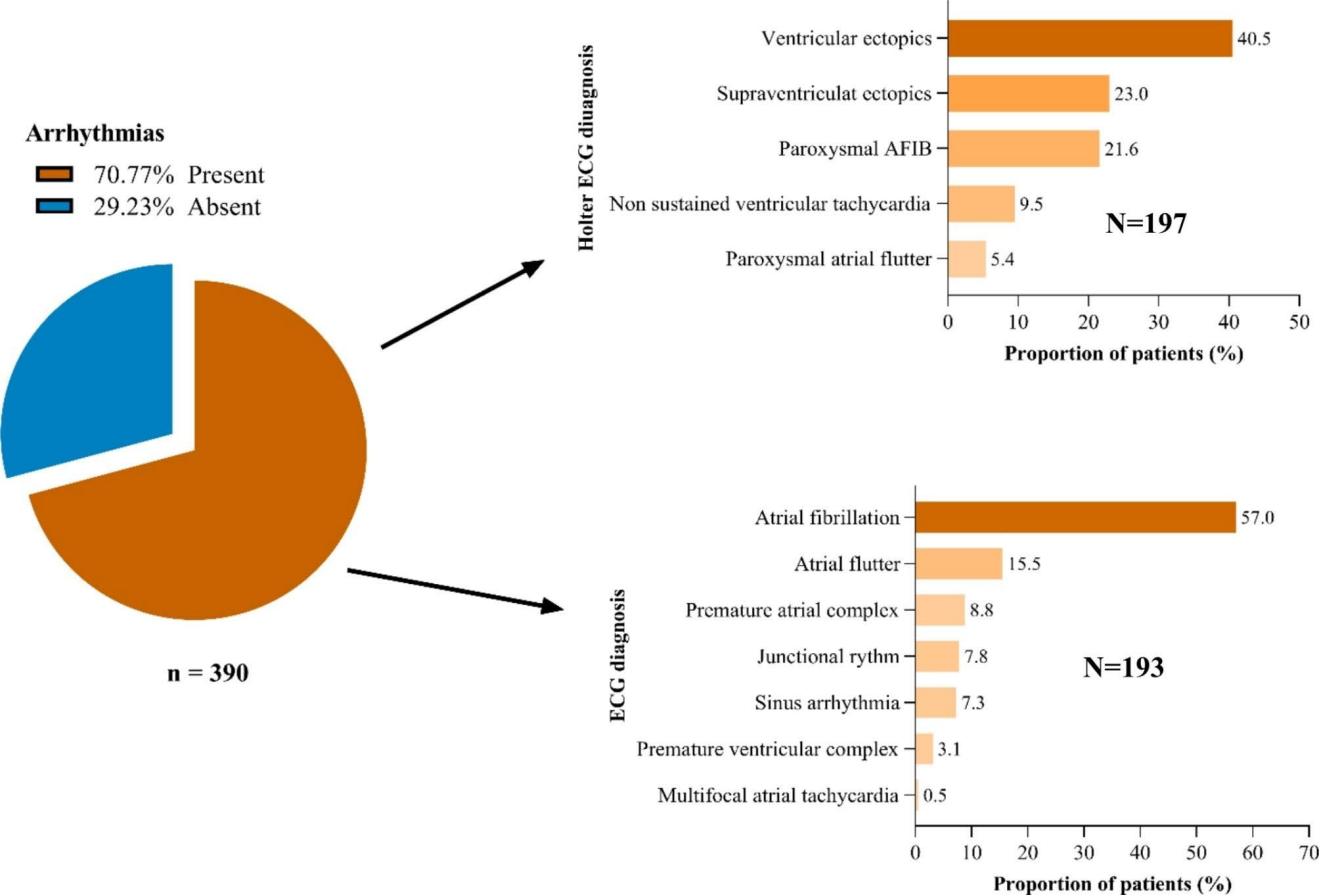
Prevalence and types of arrhythmias

The overall prevalence of arrhythmias in this study was 276/390 70.77%, of which 193/390 (49.5%) of patients had arrhythmia on resting ECG and out of 197/390 patients who did holter 88/197 (44.7%) had paroxysmal arrhythmias. The most common arrhythmia on ECG was atrial fibrillation by 57%, whereas the most common arrhythmias on holter ECG (as per our operational definition) was ventricular ectopic by 40.5%. Other arrhythmias in order of importance included atrial flutter, supraventricular ectopic, paroxysmal AF, Junctional rhythm, sinus arrhythmias, non-sustained ventricular tachycardia and paroxysmal atrial flutter. (Fig. 1)

### Socio-demographic and clinical characteristics associated with arrhythmias

Among patients with RHD, arrhythmias occurred more common in age > 60 (88.2%).The prevalence of arrhythmias was same between males and females (74.4% vs. 68.9%, p = 0.252). High proportion (76.5%) of patients with arrhythmias had comorbidities such ashypertension, diabetes mellitus and stroke, symptoms of arrhythmias (80.8%), NHYA III-IV functional class (91.9%), Tricuspid regurgitation (83.4%) and had no history of cardiac surgery (70.9%). Factors that were significantly associated with arrhythmias were age, presence of symptoms of arrhythmias, higher NYHA class, mitral stenosis, and tricuspid regurgitation as shown in Table 2.

**Table 2:** Social demographic and clinical characteristics associated with the development of arrhythmias n = 390

Variables	Yes (%)	No (%)	P - value
Age (years)			
18–30	69 (68.3)	32 (31.7)	0.014
31–50	119 (65.7)	62 (34.3)	
51–60	43 (75.4)	14 (24.6)	
> 60	45 (88.2)	6 (11.8)	
Sex			
Male	99 (74.4)	34 (25.6)	0.252
Female	177 (68.9)	80 (31.1)	
Comorbidities			
Yes	13 (76.5)	4 (23.5)	0.787
No	263 (70.5)	110 (29.5)	
Cardiac surgery			
Yes	69 (70.4)	29 (29.6)	0.928
No	207 (70.9)	85 (29.1)	
Symptoms of arrhythmias			
Symptomatic	147 (80.8)	35 (19.2)	< 0.001
Asymptomatic	129 (62.0)	79 (38.0)	
NYHA Functional Class			
NYHA I-II	140 (57.9)	102 (42.1)	< 0.001
NYHA III-IV	136 (91.9)	12 (8.1)	
Valve lesion			
Mitral stenosis	177 (77.6)	51 (22.4)	< 0.001
Mitral regurgitation	135 (73.4)	49 (26.6)	0.286
Aortic regurgitation	88 (69.8)	38 (30.2)	0.739
Tricuspid regurgitation	156 (83.4)	31 (16.6)	< 0.001

### Echocardiographic parameters associated with arrhythmias

Prevalence of arrhythmias was high among patients with decreased trans annular plane systolic excursion (TAPSE), severe left ventricular dysfunction, patients with pulmonary hypertension and those with severely dilated LA. (Table 3).

**Table 3:** Echocardiographic characteristics associated with arrhythmias n = 390

		Presence of Arrhythmia		
Variable		Yes (%)	No (%)	P-value
TAPSE				
	Normal	208 (67.1)	102 (32.9)	0.002
	Decreased	68 (85)	12 (15)	
LVEF				
	Normal	154 (65)	83 (35)	0.004
	Mild dysfunction	73 (75.3)	24 (24.7)	
	Moderate dysfunction	30 (83.3)	6 (16.7)	
	Severe dysfunction	19 (95)	1 (5)	
Evidence of Pulmonary Hypertension				
	Yes	131 (82.4)	28 (17.6)	< 0.001
	No	145 (62.8)	86 (37.2)	
Pericardial effusion				
	yes	25 (78.1)	7 (21.9)	0.340
	no	251 (70.1)	107 (29.9)	
Presence of thrombus				
	Yes	7 (58.3)	5 (41.7)	0.344
	No	269 (71.2)	109 (28.8)	
Mitral Valve area				
	Mild decreased	45 (70.3)	19 (29.7)	
	Moderate decreased	32 (86.5)	5 (13.5)	
	Severe decreased	104 (80.6)	25 (19.4)	0.116
Mean gradient				
	Mild increased	4 (80)	1 (20)	
	Moderate increased	43 (74.1)	15 (25.9)	
	Severe increased	134 (80.2)	33 (19.8)	0.572
LA diameter				
	Normal	13 (25.5)	38 (74.5)	< 0.001
	Mild dilated	23 (60.5)	15 (39.5)	
	Moderate dilated	33 (66)	17 (34)	
	Severe dilated	207 (82.5)	44 (17.5)	
LVIDd				
Normal		76 (73.8)	27 (26.2)	0.658
Mild dilated		2 (50)	2 (50)	
Moderate dilated		6 (75)	2 (25)	
Severe dilated		192 (69.8)	83 (30.2)	
LVIDs				
	Normal	171 (66.5)	86 (33.5)	0.681
	Mild dilated	28 (80)	7 (20)	
	Moderate dilated	8 (72.7)	3 (27.3)	
	Severe dilated	69 (79.3)	18 (20.7)	

### Independent factors associated with arrhythmias

Logistic regression analysis using multivariate model (Table 4) with eight factors from chi-square table. When the eight characteristics were adjusted for confounders two factors were found to be independently associated with arrhythmias, NYHA III &IV (aOR 4.67 95% CI 1.82-12.00) p < 0.001) and severe LA enlargement (aOR 7.28 95% CI 3.17–16.70) p < 0.01).

**Table 4:** Logistic regression analysis showing independent factors associated with arrhythmia

		Univariate analysis			Multivariate analysis		
Variable		cOR	95% CI	P-value	aOR	95% CI	P-value
Age groups(years)							
	18–30	1.12	0.67–1.89	0.661	0.93	0.49–1.79	0.845
	51–60	1.60	0.81–3.15	0.173	0.90	0.40–2.03	0.800
	> 61	3.91	1.58–9.66	0.003	2.18	0.76–6.21	0.145
	31–50	Ref					
Symptoms							
	symptomatic	1.63	1.23–2.16	< 0.001	1.44	0.81–2.57	0.212
	asymptomatic	Ref					
NYHA class							
	III & IV	8.26	4.34–15.71	< 0.001	4.67	1.82–12.00	0.001
	I & II	Ref					
Mitral stenosis							
	yes	2.21	1.42–3.44	< 0.001	1.106	0.59–2.06	0.750
	no	Ref					
Tricuspid regurgitation							
	yes	3.44	2.13–5.54	< 0.001	1.61	0.77–3.36	0.203
	no	Ref					
Pulmonary hypertension							
	yes	2.77	1.70–4.52	< 0.001	0.90	0.43–1.90	0.789
	no	Ref					
Left atrial diameter							
	mild enlarged	4.48	1.81–11.08	< 0.001	3.45	1.25–9.49	0.017
	moderate enlarged	5.67	2.40–13.40	< 0.001	3.23	1.20–8.69	0.021
	severe enlarged	13.75	6.77–27.94	< 0.001	7.28	3.17–16.70	< 0.001
	normal	Ref					
Left ventricular ejection fraction							
	mild dysfunction	4.48	1.81–11.08	0.001	0.95	0.49–1.84	0.875
	moderate dysfunction	5.67	2.40–13.40	< 0.001	1.59	0.46–5.49	0.464
	Severe dysfunction	13.75	6.77–27.94	< 0.001	2.73	0.32–23.56	0.361
Key: cOR: crude Odds Ratio, aOR adjusted Odds Ratio, Ref: Reference category,							

The cut off point for left atrial dilatation beyond which arrhythmias develop in our study as shown in Fig. 2 was found to be 48 mm with a sensitivity of 81.88% and positive predictive value of 84.64%.

**Fig. 2:**
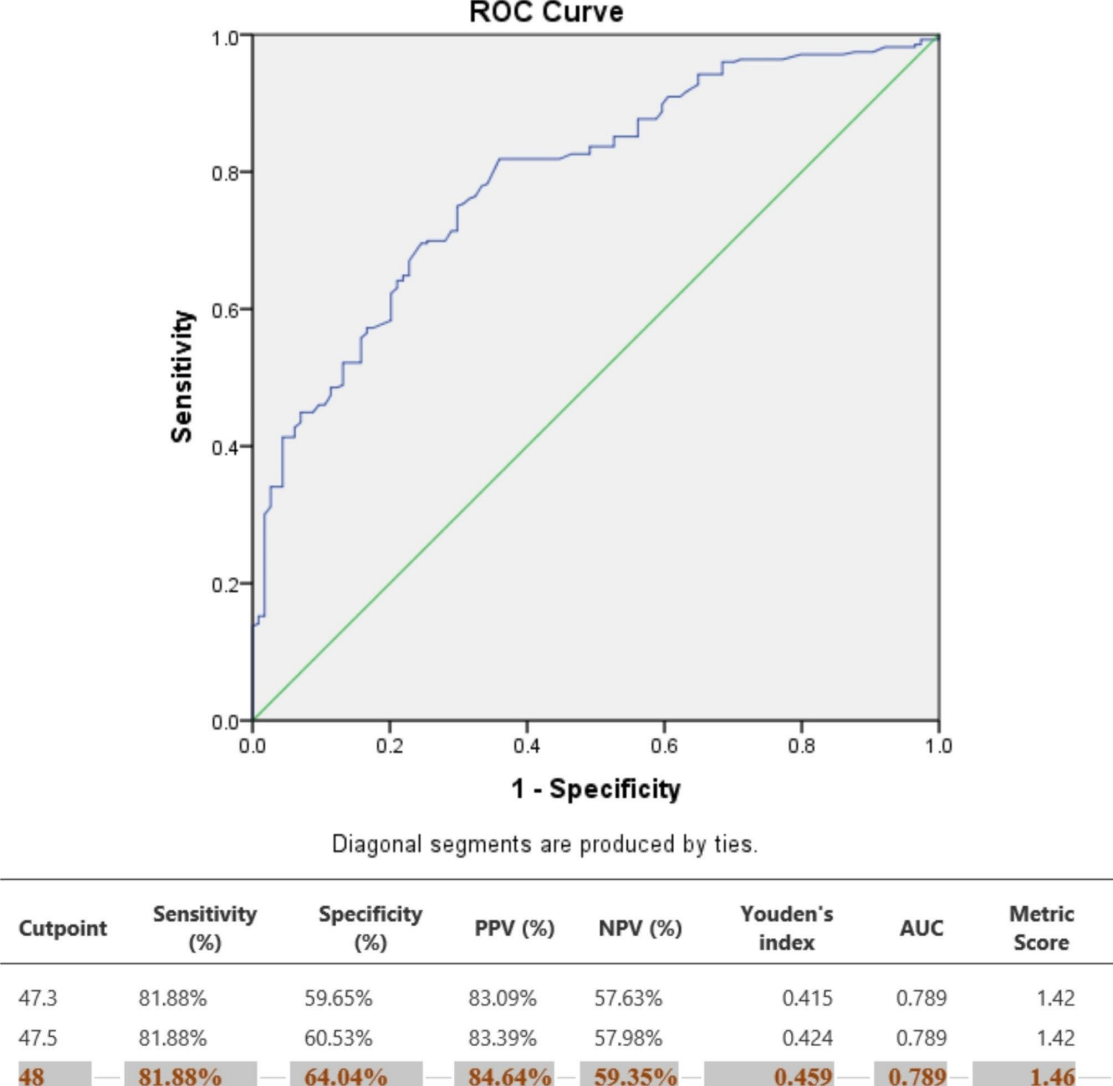
Cut off prediction valueofleft atrium sizefor arrhythmias development

## Discussion

Arrhythmias were identified in 276/390 (70.77%) of patients, with 193/390 (49.5%) having arrhythmias on the resting ECG. A 24-hour Holter monitoring was performed on 197/390 (50.5%) of the patients, and it was discovered that 88/197 (44.7%) of them experienced arrhythmias. This is in contrast with a study done by Behra S et al that found that out of 268 patients with RHD 45.5% had arrhythmias [8]. Another study by Pourafkari L et al. on factors associated with arrhythmias in rheumatic mitral stenosis, they found that arrhythmias was present in 33% of patients however in this study they only assessed for atrial fibrillation [13].

In a meta-analysis done globally among 75,637 patients with RHD it was found that 32.8% patients had arrhythmias, however this study did not assess for paroxysmal arrhythmias [14].

Bouleti C et al. found the prevalence to be 38%, however this study only involved patients who had undergone percutaneous mitral valve repair [15]. The Soweto Heart Study found the prevalence of arrhythmias to be 46.8% [7].

Types of arrhythmias in this study were AF (57%), atrial flutter (15.5%), premature atrial complexes (PAC) (8.8%), junctional rhythm (7.8%), sinus arrhythmia (7.3), PVC (3.1%) and multifocal atrial tachycardia (0.5%). Similarly, Behra S et al. found that AF was present in 37.2% of patients followed by ventricular ectopic beats in 8.9% patients, multifocal atrial tachycardia in 1.8% patients [8]. Holter monitoring in patients with mitral stenosis and sinus rhythm in a study done by Ramsdale DR et al. showed that supraventricular ectopic was in 93.6% patients, paroxysmal atrial fibrillation was present in 22.2% patients, atrial flutter was in 7.9% patients, ventricular ectopic was in 87.3% and 1 patient had non sustained supraventricular tachycardia. Overall, our findings and findings from previous studies suggest that supraventricular arrythmias are the most common arrhythmias in RHD.The variation in prevalence and types of arrhythmias is due to the use of different definitions when diagnosing arrhythmias and the device used to detect arrhythmias.

This study found that increase NYHA functional class was significantly associated with arrhythmias in both univariate and multivariate analysis. Higher NYHA functional class occurs in patients with severe valve disease and chamber dilatation which is the cornerstone for arrhythmias. Alam et al. found that association of arrhythmias with increasing NYHA class was significant for pauses, paroxysmal supraventricular tachycardia, AF, couplets, bigeminy and trigeminy [5].

In this study we found that mitral stenosis, tricuspid regurgitation, left ventricular dysfunction, pulmonary hypertension, and LA diameter were significantly associated with arrhythmias in univariate analysis. However, in multivariate analysis, only LA dilatation was significantly associated with arrhythmias. This observation is similar to that of Diker E et al. [16]. In contrast, a Meta-analysis of correlates of AF in RHD by Noubiap et al. found that mitral valve disease and tricuspid valve involvement (OR:4 95% CI 3.0-5.3) and LA dilatation (MD:8.1 mm 95% CI 5.5–10.7) was associated with AF [14]. The observed difference could be due to different methodology, study population and sample size.

In a study done in India on VHD they found that LVIDd and LVIDs was significantly associated with arrhythmias however, this finding was only for mitral regurgitation, aortic regurgitation and aortic stenosis lesions, when the parameters where compared to multivalvular involvement it appeared not to be significantly associated with arrhythmias [8]. Diker E et al. found that LVIDd and LVIDs were not statically significant associated with arrhythmias, this finding is similar to the finding in our study.

In receiver operating curve we found that the critical point beyond which arrhythmias develop was LA diameter > 48 mm. This is not similar to that of S Behra et al., they found that the critical point for LA dilatation of > 43 mm. This could be due different ethnicity among the two studies.

In our study mitral valve area and mean gradient was not significantly associated with arrhythmias this contrasts with S Behra et al., they found that mitral valve area and mean gradient to be significant associated with arrhythmias in univariate model, however in their multivariate analysis it was not significant. Our finding was like that of Diker E et al., MVA did not show any correlation as a predictor for AF.

The recentness of this study is that no current data is available in Tanzania that characterizes arrhythmias among RHD patients; this study shows that LA dilatation is a good predictor of arrhythmias and thus can be used in risk stratification among these patients. Holter ECG has shown to be an important tool in detecting arrhythmias among patients with normal sinus rhythm in resting ECG.

### Limitation of the study

The inherent nature of study design in using 24 h holter ECG might have led to underestimation of some arrhythmias which could be life threatening, more findings would have been obtained if more than 24 h of holter monitoring had been used.

## Conclusion

We found a high prevalence of arrhythmias among patients with RHD. The independent predictors of arrhythmias were LA dilatation and NYHA functional class III-IV. LA diameter of greater than 48 mm is a predictor of arrhythmias. We recommend holter monitoring in RHD patients with sinus rhythm on resting ECG who present with dilated LA of above 48 mm and higher NYHA functional class.

## Data Availability

The datasets generated and analyzed during the current study are available from the corresponding author on reasonable request and materials.

## Abbreviations

RHD: rheumatic heart disease
ECG: electrocardiography
JKCI: Jakaya Kikwete Cardiac Institute
NYHA: New York Heart ECHO:echocardiography
LA: left atrium
PVC: Premature ventricular contraction
AF: Atrial fibrillation
IQR: Interquartile range
aOR: adjusted odds ratio

## References

[1] Singh M, Malhotra P, Thakur JS (1997). Rheumatic heart disease in developing countries [16]. Lancet.

[2] Itzikowitz G, Prendergast EA, Prendergast BD, Zühlke L. Acute rheumatic fever and rheumatic heart disease. Hear Valve Dis State Art. 2019;(November 2001):163–75.

[3] Watkins DA, Beaton AZ, Carapetis JR, Karthikeyan G, Mayosi BM, Wyber R (2018). Rheumatic Heart Disease Worldwide: JACC Scientific Expert Panel. J Am Coll Cardiol.

[4] Watkins DA, Johnson CO, Colquhoun SM, Karthikeyan G, Beaton A, Bukhman G (2017). Global, Regional, and National Burden of Rheumatic Heart Disease, 1990–2015. N Engl J Med.

[5] Alam S. Correlation of paroxysmal and persistent cardiac arrhythmias with clinical and echocardiographic parameters in patients of rheumatic fever and Rheumatic Heart Disease. 2020;(June 2019).

[6] Papadopoulos CH, Oikonomidis D, Lazaris E, Nihoyannopoulos P. Echocardiography and cardiac arrhythmias. Hell J Cardiol [Internet]. 2018;59(3):140–9. Available from: 10.1016/j.hjc.2017.11.017. 10.1016/j.hjc.2017.11.017 29203161

[7] Sliwa K, Wilkinson D, Hansen C, Ntyintyane L, Tibazarwa K, Becker A (2008). Spectrum of heart disease and risk factors in a black urban population in South Africa (the heart of Soweto Study): a cohort study. Lancet.

[8] Behra SS, Anil Kumar AVS, Singh H, Satyanand K. To study the prevalence of arrhythmias in valvular heart disease and their correlation with echocardiographic variables. J Clin Diagnostic Res. 2018;12(11).

[9] Okello E, Longenecker CT, Beaton A, Kamya MR, Lwabi P. Rheumatic heart disease in Uganda: Predictors of morbidity and mortality one year after presentation. BMC Cardiovasc Disord [Internet]. 2017;17(1):1–10. Available from: 10.1186/s12872-016-0451-8. 10.1186/s12872-016-0451-8 PMC5219796 28061759

[10] Lang RM, Badano LP, Mor-avi V, Afilalo J, Armstrong A, Ernande L et al. Recommendations for Cardiac Chamber Quantification by Echocardiography in Adults: An Update from the American Society of Echocardiography and the European Association of Cardiovascular Imaging. J Am Soc Echocardiogr [Internet]. 2015;28(1):1–39.e14. Available from: 10.1016/j.echo.2014.10.003. 10.1016/j.echo.2014.10.003 25559473

[11] Mitchell C, Rahko PS, Blauwet LA, Canaday B, Finstuen JA, Foster MC et al. Guidelines for Performing a Comprehensive Transthoracic Echocardiographic Examination in Adults: Recommendations from the American Society of Echocardiography. J Am Soc Echocardiogr [Internet]. 2019;32(1):1–64. Available from: 10.1016/j.echo.2018.06.004. 10.1016/j.echo.2018.06.004 30282592

[12] Suliman A, Kapur A, Archbold RA, Ranjadayalan K, Phil M, Timmis AD. Limited clinical utility of Holter Monitoring in patients with palpitations or altered consciousness: analysis of 8973 recordings in 7394 patients. 2008;(0):39–43. 10.1111/j.1542-474X.2007.00199.x PMC6932525 18234005

[13] Pourafkari L, Ghaffari S, Bancroft GR, Tajlil A, Nader ND, Cardiovascular A. Asian Cardiovasc Thorac Annals. 2014. 10.1177/0218492314530134 24696100

[14] Noubiap JJ, Nyaga UF, Ndoadoumgue AL, Nkeck R, Ngouo A, Bigna JJ (2020). Meta-analysis of the incidence, prevalence, and correlates of Atrial Fibrillation in. Rheumatic Heart Disease.

[15] Bouleti C, Iung B, Laouénan C, Himbert D, Brochet E, Messika-Zeitoun D (2012). Late results of percutaneous mitral commissurotomy up to 20 years: development and validation of a risk score predicting late functional results from a series of 912 patients. Circulation.

[16] Harrison H, Holm J (1995). Prevalence and predictors of Atrial Fibrillation in Rheumatic Valvular Heart Disease. Am J Cardiol.

